# High-resolution climate projection dataset based on CMIP6 for Peru and Ecuador: BASD-CMIP6-PE

**DOI:** 10.1038/s41597-023-02863-z

**Published:** 2024-01-05

**Authors:** Carlos Antonio Fernandez-Palomino, Fred F. Hattermann, Valentina Krysanova, Fiorella Vega-Jácome, Christoph Menz, Stephanie Gleixner, Axel Bronstert

**Affiliations:** 1 Potsdam Institute for Climate Impact Research (PIK), Member of the Leibniz Association, PO Box 60 12 03, D-14412 Potsdam, Germany; 2 Institute of Environmental Science and Geography, University of Potsdam, Potsdam, Germany

**Keywords:** Hydrology, Water resources

## Abstract

Here, we present BASD-CMIP6-PE, a high-resolution (1d, 10 km) climate dataset for Peru and Ecuador based on the bias-adjusted and statistically downscaled CMIP6 climate projections of 10 GCMs. This dataset includes both historical simulations (1850–2014) and future projections (2015–2100) for precipitation and minimum, mean, and maximum temperature under three Shared Socioeconomic Pathways (SSP1-2.6, SSP3-7.0, and SSP5-8.5). The BASD-CMIP6-PE climate data were generated using the trend-preserving Bias Adjustment and Statistical Downscaling (BASD) method. The BASD performance was evaluated using observational data and through hydrological modeling across Peruvian and Ecuadorian river basins in the historical period. Results demonstrated that BASD significantly reduced biases between CMIP6-GCM simulations and observational data, enhancing long-term statistical representations, including mean and extreme values, and seasonal patterns. Furthermore, the hydrological evaluation highlighted the appropriateness of adjusted GCM simulations for simulating streamflow, including mean, low, and high flows. These findings underscore the reliability of BASD-CMIP6-PE in assessing regional climate change impacts on agriculture, water resources, and hydrological extremes.

## Background & Summary

Reliable hydro-climate data are essential for understanding the effects of observed and projected climate change on social and natural systems and developing effective adaptation and mitigation strategies. Several global and regional observation datasets exist at different temporal and spatial resolutions^1–10^. At the regional scale, the Servicio Nacional de Meteorología e Hidrología del Perú (SENAMHI) has developed the Peruvian Interpolated data of Climatological and Hydrological Observations (PISCO). The PISCO dataset includes precipitation^7^, maximum and minimum temperature^5^, and reference evapotranspiration^6^ data. SENAMHI uses this dataset for drought and flood monitoring at the countrywide level of Peru. Recently a new hydrologically corrected daily precipitation dataset, called RAIN4PE (Rain for Peru and Ecuador), was developed and is available^10,11^. RAIN4PE has proved to be superior to other existing precipitation datasets such as CHIRP^2^, CHIRPS^2^, ERA5^9^, MSWEP^3,4^, and PISCO-precipitation^7^ for hydrometeorological applications and suggested to be a basis for bias adjustment of Global Climate Models (GCMs) output in Peru and Ecuador^10^.

GCMs have become important tools for historical climate simulation and future climate projection^12^. As part of the Coupled Model Intercomparison Project (CMIP) of the World Climate Research Programme (WRCP), GCMs output has contributed to the assessment reports produced by the Intergovernmental Panel on Climate Change (IPCC)^12–14^. Output from the latest generation of GCMs participating in the sixth phase of the CMIP (CMIP6) is now available^15^. CMIP6 models have a better spatial resolution (~100 km in the horizontal dimension) and physical process representation than earlier generations, as well as better simulation of recent mean climate compared to the previous CMIP phases^16^. Nevertheless, such a resolution is still coarse for regional and local management decisions, which need more detailed climate information. Namely, such coarse-resolution data are useless for providing a reliable base for real-world water management, particularly related to extreme hydrological conditions^17^. Moreover, even the latest generation of GCMs shows substantial biases^18–20^. Therefore, it is important to bias-adjust and downscale the raw GCM outputs to produce reliable climate simulations and projections for finer-scale impact studies. To date, few studies have performed the bias adjustment and downscaling of the output of CMIP6 models, e.g., at a global scale^21–25^, for South Asia^26^, and for Brazil^27^. To the best of our knowledge, there is currently no gridded dataset based on CMIP6 results bias-adjusted and downscaled for Peru and Ecuador using reliable reference data from the local observation datasets. To close this gap, we generated the new high-resolution climate dataset BASD-CMIP6-PE based on the bias-adjusted and statistically downscaled CMIP6 climate projections over Peru and Ecuador.

The BASD-CMIP6-PE dataset was generated using the trend-preserving Bias Adjustment and Statistical Downscaling (BASD) method^28,29^. BASD effectively reduced biases between CMIP6-GCM simulations and observational data, resulting in improved representations of long-term statistical properties, including mean and extreme values, as well as seasonal patterns. BASD also demonstrated its capability to approximately preserve the projected trends and the intermodel spreads of climate variables in future climate scenarios.

A hydrological evaluation, which compared raw and adjusted GCM simulations through hydrological modeling, provided additional support for the appropriateness of adjusted GCM simulations in simulating streamflow, including mean, low, and high flows.

These advantages underscore the dataset’s reliability for assessing regional climate change impacts on agriculture, water resources, and hydrological extremes, thereby supporting the development of comprehensive adaptation strategies. Notably, the BASD-CMIP6-PE dataset has already played a pivotal role in conducting the first-ever investigation into projected future changes in various components of the regional hydrological cycle and hydrological extremes across Peru, including the analysis of transboundary river catchments^30^.

## Methods

### Study area

The study area covers Peru and Ecuador. The new BASD-CMIP6-PE dataset is generated for the land surface between 19°S–2°N and 82°–67°W which matches the observational data domain of the RAIN4PE and PISCO datasets. This region features complex hydroclimatic patterns arising from its diverse climate zones and the Andes Cordillera, which serves as a topographic barrier separating the cold, arid eastern Pacific from the warm, humid Amazon. These patterns result from the interplay of large-scale factors (e.g., latitudinal migration of the Atlantic Intertropical Convergence Zone, South American Monsoon Systems, marine currents, Bolivian High) and local circulation patterns (e.g., upslope and downslope moisture transport), in conjunction with the complex Andean orography^31–34^. El Niño-Southern Oscillation (ENSO) also significantly influences interannual hydroclimatic conditions in the Andes^35^.

### Climate simulation data

Daily climate model output data for precipitation (pr) and minimum (tasmin), mean (tas), and maximum (tasmax) temperature were obtained from the CMIP6 ensemble^15^ for 10 GCMs (Table 1). Data were obtained for the historical simulation (1850–2014) and future projections (2015–2100), with projections run under SSP1-2.6, SSP3-7.0, and SSP5-8.5 scenarios^36^. These ten models were also used by phase 3b of the Inter-Sectoral Impact Model Intercomparison Project (ISIMIP3b) for climate impact assessment studies^24,25^. In terms of climate sensitivity (i.e., magnitude of the warming signal at the end of the century), the selected models are considered an appropriate choice since they approximately cover the full range of CMIP6 projections as they include models with low (GFDL-ESM4, MPI-ESM1-2-HR, MRI-ESM2-0) and high (IPSL-CM6A-LR, UKESM1-0-LL) climate sensitivity^37^. The selected three greenhouse gas emissions scenarios span from one with mitigation policy (SSP1-2.6) to one without mitigation (SSP5-8.5) to sample future climate uncertainty from anthropogenic forcing. The SSP1-2.6 is close to the Paris Agreement goal, where global warming is limited to 2 °C above pre-industrial levels. The scenario is characterized by declining greenhouse gas (GHG) emissions to net zero until 2050, followed by varying levels of net negative CO2 emissions. The SSP3-7.0 scenario is a high and the SSP5-8.5 a high-end global warming scenario with continuing high fossil fuel development throughout the 21st century and consequently strong increases in GHG emissions. For a detailed description of SSPs scenarios, refer to the Sixth Assessment Report of the IPCC^14^.

**Table 1:** List of the 10 CMIP6 models used in this study.

No.	Model	Resolution (lon. by lat.)	Member	Citation
1	CanESM5	2.8° × 2.8°	r1i1p1f1	^59^
2	IPSL–CM6A–LR	2.5° × 1.3°	r1i1p1f1	^60^
3	UKESM1–0–LL	1.9° × 1.3°	r1i1p1f2	^61^
4	CNRM–CM6–1	1.4° × 1.4°	r1i1p1f2	^62^
5	CNRM–ESM2–1	1.4° × 1.4°	r1i1p1f2	^63^
6	MIROC6	1.4° × 1.4°	r1i1p1f1	^64^
7	GFDL–ESM4	1.3° × 1°	r1i1p1f1	^65^
8	MRI–ESM2–0	1.1° × 1.1°	r1i1p1f1	^66^
9	MPI–ESM1–2–HR	0.9° × 0.9°	r1i1p1f1	^67^
10	EC–Earth3	0.7° × 0.7°	r1i1p1f1	^68^

The models are ordered from low to high spatial resolution.

### Climate observation

The observational reference datasets used for the evaluation, bias adjustment and statistical downscaling (BASD) of CMIP6 climate simulations are listed in Table 2. Data of precipitation (pr) were obtained from the RAIN4PE dataset^10,11^, minimum (tasmin) and maximum (tasmax) temperature from the SENAMHI-PISCO dataset^5^, and mean temperature (tas) was estimated as the average of tasmin and tasmax. RAIN4PE precipitation data (available for 1981–2015) are generated by merging multisource precipitation data (satellite, reanalysis, and ground-based precipitation from 804 gauges) with surface elevation using the random forest method. Additionally, total precipitation was adjusted using streamflow data through the reverse hydrology method for catchments influenced by fog/cloud water input, such as páramo and montane watersheds. The PISCO temperature data (available for 1981–2016) are generated by merging information from 178 observed climate stations, satellite-derived surface temperatures, and topographic variables. The data integrate spatially gridded estimates of normal climate (estimated using weighted regression Kriging) with daily anomalies (estimated using regression splines). These observational datasets have been validated by simulating streamflow through hydrological modeling for the Peruvian and Ecuadorian catchments^10^.

**Table 2:** Climate observed data used in this study.

Variable	Resolution	Description and source
Precipitation	Daily/0.1°	Rain for Peru and Ecuador (RAIN4PE^10,11^)
Temperature	Daily/0.1°	Maximum and minimum temperature data for Peru^5^ as provided by SENAMHI (ftp://publi_dgh2:123456@ftp.senamhi.gob.pe/)

### Bias adjustment and statistical downscaling

The software used for BASD is ISIMIP3BASD v2.5^29^ which implements the BASD method described in Lange^28^. In ISIMIP3, this method was applied to generate bias-adjusted and downscaled CMIP6 projections, utilizing the global observational dataset W5E5 (available at a spatial resolution of 0.5°) as reference data^24,25,38^. In our study, we adopted a different approach, employing highly reliable, region-specific, high-resolution datasets, namely PISCO-temperature and RAIN4PE precipitation, to develop BASD-CMIP6-PE. The BASD method uses 1) a trend-preserving quantile mapping approach to bias-adjust climate simulation data at their original spatial resolution using spatially aggregated climate observation data, and 2) a stochastic statistical downscaling approach to increase their spatial resolution using climate observation data, which consequently have to be available with the higher resolution. Note that bias adjustment (BA) and statistical downscaling (SD) are applied after one another and not together. Further details on the BASD method are given in Lange^28^.

**Fig. 1:**
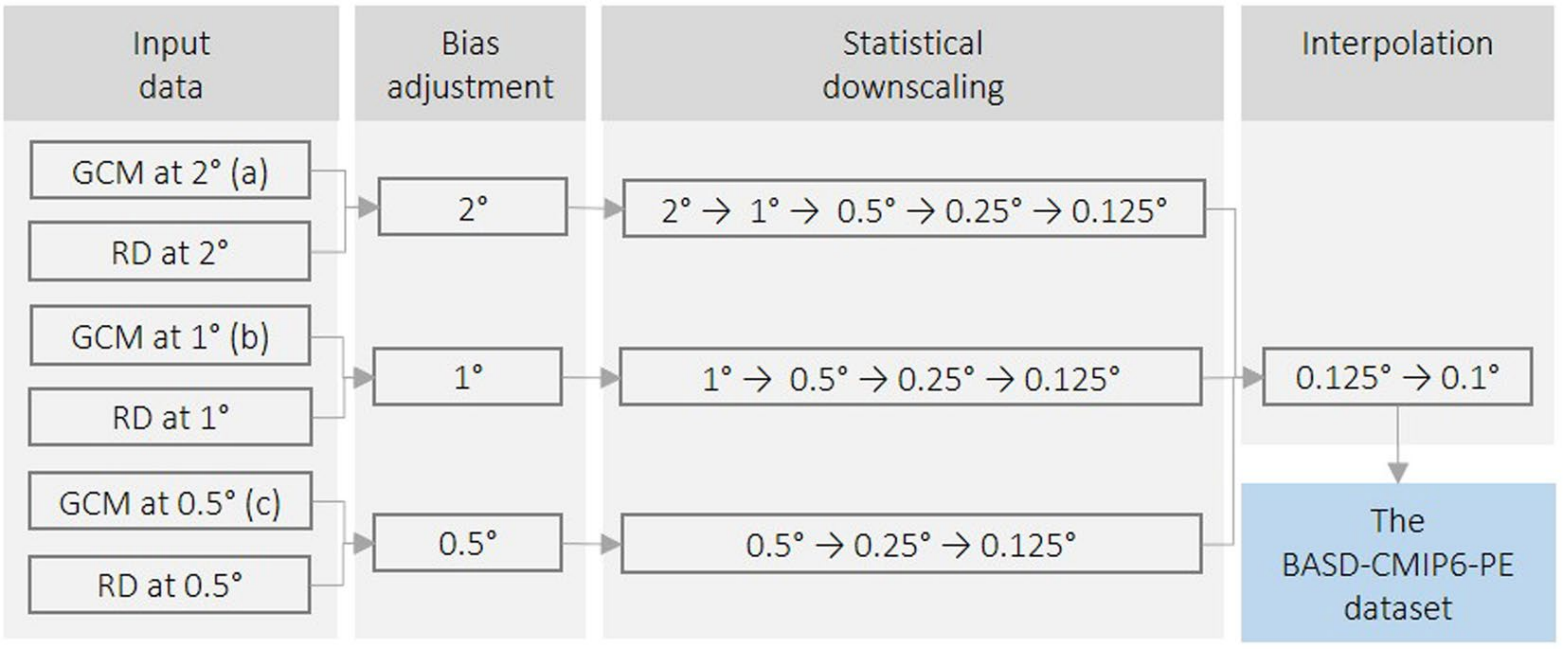
Flow chart for the BASD-CMIP6-PE dataset variables (pr, tasmin, tas, tasmax) for 10 GCMs. The bias adjustment was applied at: (**a**) 2° for CanESM5, IPSL-CM6A-LR, and UKESM1-0-LL; (**b**) 1° for CNRM-CM6-1, CNRM-ESM2-1, GFDL-ESM4, MIROC6, MPI-ESM1-2-HR, and MRI-ESM2-0; and (**c**) 0.5° for EC-Earth3. GCM (RD) is Global Climate Model (reference) data. Note that RD are aggregated observation data.

Figure 1 shows the bias adjustment and downscaling strategy to generate the BASD-CMIP6-PE dataset following the ISIMIP protocol^37^. This dataset includes both historical simulations (1850–2014) and future projections (2015–2100) for four variables (pr, tasmin, tas, tasmax) and three future CMIP6 scenarios (SSP1-2.6, SSP3-7.0, SSP5-8.5) for 10 GCMs. To apply the ISIMIP3BASD method, the original CMIP6 output (observational data) was interpolated (aggregated) onto regular latitude-longitude grids with 0.5°, 1.0° or 2.0° resolutions using the first-order conservative remapping^39^; Fig. 1 shows which resolution was used for each climate model. The interpolated CMIP6 data were bias-adjusted using the respective aggregated observational data and then downscaled in multiple steps. Finally, the downscaled data (0.125°) were interpolated onto 0.1° with the first-order conservative remapping method to match the spatial resolution of the observational data. This interpolation was carried out instead of downscaling due to the small resolution difference from 0.125° to 0.1°.

The training period used for bias adjustment and statistical downscaling was 1981–2014 (34 years), which was constrained by the availability of observed data and historical CMIP6 simulations. After training, we applied the bias adjustment and downscaling on the climate simulations over historical and future periods using contiguous 36-yr segments. This decomposition is recommended to keep a similar sample size in the training and application steps. Bias adjustment was applied using a running window with a width of 31 days and moved over the annual cycle in the step of 1 day, where results for the central day of each window constitute the overall result. This application pattern aimed to improve the annual cycle representation and reduce discontinuities at time window edges, as suggested in previous studies^40–43^.

### Evaluation approach

We conducted a comprehensive evaluation of the BASD method’s performance and the reliability of the BASD-CMIP6-PE dataset for the historical period. This assessment included comparing the simulations from both unadjusted and adjusted CMIP6 models with observational data and employing hydrological modeling. Our primary focus was on evaluation of the models’ capability in representing long-term statistical aspects, including mean values, extreme values, and the mean annual cycle. Moreover, we examined whether the BASD method influenced the preservation or alteration of the projected GCM trends (or changes) and the inter-model spread.

Model simulations and observations were compared using widely applied climate modeling statistics (e.g., mean error, correlation coefficient) and the Taylor diagram^44^ for the overlapping time period 1981–2014. Mean error was used to show biases in GCM data, while the correlation coefficient assessed models’ capability to represent the mean annual cycle of observations, a crucial aspect for hydrological modeling purposes. The Taylor diagram was used to summarize the performance of both unadjusted and adjusted CMIP6 models in simulating long-term climatological spatial fields, including mean and extreme values of precipitation and temperature across the entire study domain. According to the Taylor diagram, the closer the model points are to the reference point, the better the model performance is with relatively high correlation and low standard deviation and root-mean-square error (RMSE) values. Extreme values were determined by calculating the 95th percentile for precipitation and maximum temperature and the 5th percentile for minimum temperature. Note that simulations and reference data were conservatively interpolated to a 2° × 2° latitude-longitude grid to facilitate the comparison.

The reliability of both raw GCM data and BASD-CMIP6-PE dataset for describing the climatology of climate variables (pr, tas, tasmax, and tasmin) was also evaluated through hydrological modeling since the response of the watershed’s flow is primarily driven by the variations in precipitation and temperatures. This approach was used in recent years to evaluate gauge-corrected precipitation datasets in data-scarce regions^10,45–47^, as well as raw and bias-adjusted GCM simulation data^48^.

Hydrological simulations were performed using the Soil and Water Assessment Tool (SWAT) model^49^. SWAT is one of the world’s most widely used ecohydrological models^50,51^ and it has been successfully applied for ecohydrological modeling of Andean and Amazonian catchments in Peru and Ecuador^10,52^. SWAT is a process-oriented, semi-distributed, and time-continuous river basin model applied to simulate hydrological processes as well as vegetation dynamics, nutrients, pesticides, and sediment loads within a basin^49,53^. We used the SWAT model that was set up for the Peruvian and Ecuadorian watersheds (total of 1 638 793 km^2^, including 2675 river segments), calibrated and validated over 72 stream gauges in our previous study^10^. It was forced by the observational reference climate data listed in Table 2 and was proven to represent well the water budget closure of catchments as well as discharge dynamics, including mean, low and high flows^10^. In this study, the SWAT model was run for 1981–2014 to derive the following streamflow series for the hydrological evaluation for a selected period (1984–2014):

- Qref, streamflow simulated by SWAT driven by the reference climate data listed in Table 2,
- Qgcm, the ensemble mean of the streamflow series simulated by SWAT employing raw GCM data (pr, tasmin, and tasmax) from 10 models, and
- Qbasd, the ensemble mean of the streamflow series simulated by SWAT using BASD-CMIP6-PE climate data (pr, tasmin, and tasmax) from 10 models.

The comparison between Qraw (Qbasd) and Qref reflects the reliability of the raw GCM data (BASD-CMIP6-PE dataset). For that, we used various comparison metrics based on hydrological signatures and hydrograph goodness of fit (Table 3), which were calculated using the daily values of the seasonal streamflow for the 1984–2014 period. The modified Kling-Gupta efficiency (KGE) and percent bias (PBIAS) were used for assessing model skills in representing general discharge dynamics and over or underestimation tendencies, respectively; and percent biases in flow duration curve (FDC) low segment volume (S_low_) and FDC high segment volume (S_high_) for low flows and high flows, respectively. In this multicriteria evaluation, all aspects of the FDC and hydrographs are assessed, which is important for assessing the reliability of climate simulation data for hydroclimatic applications, including extremes (floods and low flows). The best-fit value for PBIAS, S_low_, and S_high_ is 0, and the best fit for KGE is 1.

**Table 3:** Statistical metrics and hydrological signatures.

Criterion (reference)	Equation	Description
*Discharge-related performance measures*		
Kling-Gupta efficiency^69,70^	$KGE=\sqrt{{\left(r-1\right)}^{2}+{\left(\beta -1\right)}^{2}+{\left(\gamma -1\right)}^{2}}$	r is the Pearson product-moment correlation coefficient and beta (gamma) indicates the bias (relative dispersion) between reference and simulated flows
Percent bias^71^	$PBIAS=\frac{{\sum }_{i=1}^{n}\left({S}_{i}-{R}_{i}\right)}{{\sum }_{i=1}^{n}{R}_{i}}\times 100$	*n* is the number of observations under evaluation
*Signature measures based on the flow duration curve (FDC)*		
Percent bias in FDC high segment volume^72^	${S}_{high}=\frac{{\sum }_{h=1}^{H}\left({S}_{h}-{R}_{h}\right)\times 100}{{\sum }_{h=1}^{H}{R}_{h}}$	*h* = *1, 2,…, H* are flow indices located within the high flow segment (0–5% flow EP)
Percent bias in FDC low segment volume^72^	${S}_{low}=\frac{{\sum }_{l=1}^{L}\left({S}_{l}-{R}_{l}\right)\times 100}{{\sum }_{l=1}^{L}{R}_{l}}$	*l* = *1, 2,…, L* are flow indices located within the low flow segment (95–100% flow EP)

Here, R and S are the reference and simulated flow (m^3^ s^−1^), respectively; EP is exceedance probability; H and L are the indices of the minimum flow of the high flow and low flow segments, respectively.

## Data Records

The BASD-CMIP6-PE dataset^54^ is freely available under the CC BY 4.0 license at 10.5880/pik.2023.001. BASD-CMIP6-PE provides bias-adjusted and statistically downscaled CMIP6 climate projections, encompassing four meteorological variables: precipitation (pr), minimum temperature (tasmin), mean temperature (tas), and maximum temperature (tasmax). These data cover both the historical period (1850–2014) and future projections (2015–2100) under three different CMIP6 experiments (SSP1-2.6, SSP3-7.0, and SSP5-8.5) for 10 CMIP6-GCMs. Precipitation data is reported in millimeters (mm), while temperature data is presented in degrees Celsius (°C). The total size of the dataset is 374 GB.

The BASD-CMIP6-PE dataset is organized within a “daily” folder, denoting its availability at a daily temporal resolution. Within this directory, four subfolders are present: “historical” containing historical data, “ssp126” for SSP1-2.6, “ssp370” for SSP3-7.0, and “ssp585” for SSP5-8.5. Each of these subfolders further includes ten distinct folders, corresponding to different GCMs: CanESM5, IPSL–CM6A–LR, UKESM1–0–LL, CNRM–CM6–1, CNRM–ESM2–1, MIROC6, GFDL–ESM4, MRI–ESM2–0, MPI–ESM1–2–HR, and EC–Earth3. These folders store the data in the NetCDF format arranged by model, model member, experiment, variable, temporal resolution, and subset period, resulting in file names like “canesm5_r1i1p1f1_ssp126_pr_daily_2015_2020.nc”.

## Technical Validation

### Comparison of unadjusted and adjusted CMIP6 models for the historical period

#### Mean climate and seasonality

**Fig. 2:**
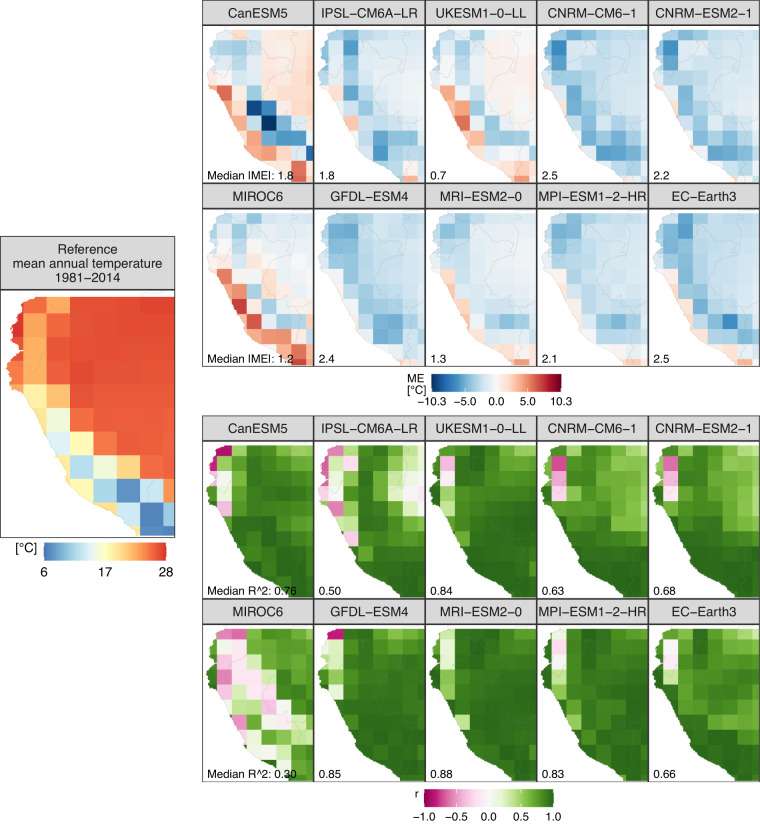
Performance of the unadjusted CMIP6 models in simulating mean temperature compared with reference temperature data from PISCO-temperature for the 1981–2014 period. ME is the mean error, r is Pearson’s correlation coefficient, and R^2^ is the coefficient of determination. r and R^2^ show the agreement between the simulated and observed mean annual temperature cycle.

**Fig. 3:**
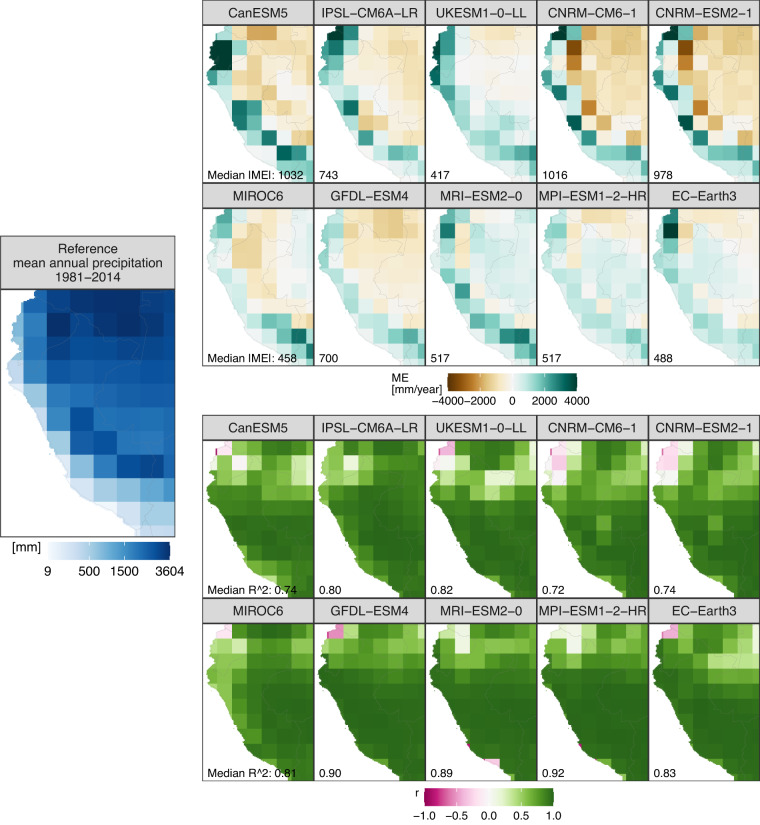
Performance of the unadjusted CMIP6 models in simulating precipitation compared with reference precipitation data from RAIN4PE for the 1981–2014 period. ME is the mean error, r is Pearson’s correlation coefficient, and R^2^ is the coefficient of determination. r and R^2^ show the agreement between the simulated and observed mean annual precipitation cycle.

**Fig. 4:**
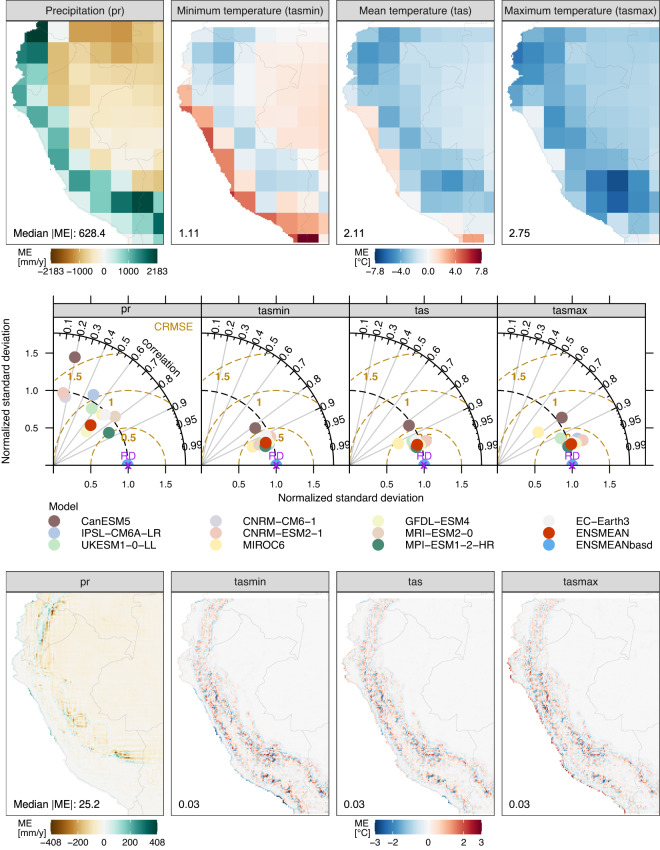
Mean Errors [ME] in (Top) unadjusted [ENSMEAN] and (Bottom) adjusted [ENSMEANbasd] CMIP6 multimodel ensemble means, and (Middle) Taylor diagrams, both comparing simulated and reference climate means for 1981–2014. Adjusted models were excluded from the Taylor diagrams as they closely match ENSMEANbasd and the reference data. The bottom panel displays ME in ENSMEANbasd, computed by comparing BASD-CMIP6-PE and observational data at a 0.1° spatial resolution.

Outputs of the atmospheric variables (pr, tasmin, tas, and tasmax) obtained from CMIP6-GCMs exhibit biases (Figs. 2–4) and limitations in capturing the mean annual cycle (Figs. 2, 3). Figure 4 (top panel) shows that CMIP6 models are generally biased cold (warm) for tas and tasmax (tasmin) over the Andes (Peruvian coastal areas) and tend to overestimate (underestimate) pr over the Andes (Amazon lowlands). Models simulate better the annual cycle of precipitation (Fig. 3) than temperature (Fig. 2). However, models cannot capture the annual cycle of pr over Ecuador and northwest Amazon (Fig. 3). This is critical as unadjusted data from models are useless, for example, for evaluating the hydrological impact of Peruvian and Ecuadorian catchments under climate change. The Taylor diagrams (Fig. 4, middle panel) show that the GCMs simulate better the spatial patterns of temperature (tasmin, tas, and tasmax) than the precipitation patterns. The models simulate a realistic spatial variability (standard deviation similar to that of reference) of precipitation, but the correlation between the spatial patterns is weak for most models (between 0.13 and 0.86). In the same Fig. 4 (middle panel), the Taylor diagram component values (correlation, standard deviation, and RMSE) indicate that the MPI-ESM1-2-HR (CanESM5) model has the best (worst) performance in simulating all atmospheric variables analyzed in this study. This distinction is emphasized by the general proximity of MPI-ESM1-2-HR points to the reference point, while CanESM5 points are situated farther away.

Overall, our results reveal that CMIP6 simulations (pr, tas, tasmin, and tasmax) are biased when compared to reference datasets over Peru and Ecuador, with larger biases over the Andes (Figs. 2–4). Such CMIP6 biases were also reported in previous studies^18,55–57^. The precipitation overestimation is likely related to the too-pronounced double ITCZ in the models, a complex error in models produced by anomalous warming over the southern tropical Pacific in association with a misrepresentation of ocean-atmosphere couplings^57,58^. Our results also reveal the limitation of CMIP6 models to reproducing the annual cycle of temperature and precipitation (Figs. 2, 3). CMIP6 models simulate the annual precipitation cycle better over regions with the strongest rainfall seasonality, such as the Peruvian Andes and lowlands, and poorly over equatorial regions, such as Ecuador and the northwest Amazon (Fig. 3). Poor representation of annual precipitation cycle by CMIP6 models was also reported over Colombia^18^ and Northern Amazon^19,20^. Our results show significant limitations of CMIP6 models in reproducing regional climate features over equatorial regions and terrains with complex topography, such as the Andes. Further improvements of these models are necessary to permit their usage in impact studies.

To exclude the aforementioned limitations in climate model outputs, we applied the BASD method in order to adjust biases, increase spatial resolution, and improve representation of the annual cycle of atmospheric variables. The comparison of the ensemble mean of unadjusted (ENSMEAN) and adjusted (ENSMEANbasd) outputs of 10 GCMs shows that the BASD method largely reduced the biases in the GCM data for all climate variables (pr, tasmin, tas, and tasmax) as shown in Fig. 4 (middle panel). However, there are remaining biases after the application of BASD at the spatial resolution of the reference datasets at 0.1° (Fig. 4, bottom panel). Small biases of precipitation remain over precipitation hotpots and small temperature biases (tasmin, tas, and tasmax) along the Andes. These results indicate that the application of BASD to the output of CMIP6 models is challenging in terrains with complex topography, such as the Andes. Despite some remaining biases, the purpose of the method - to create a dataset suitable for hydrological modeling - has been achieved, as the following hydrological section demonstrates.

#### Extreme values

**Fig. 5:**
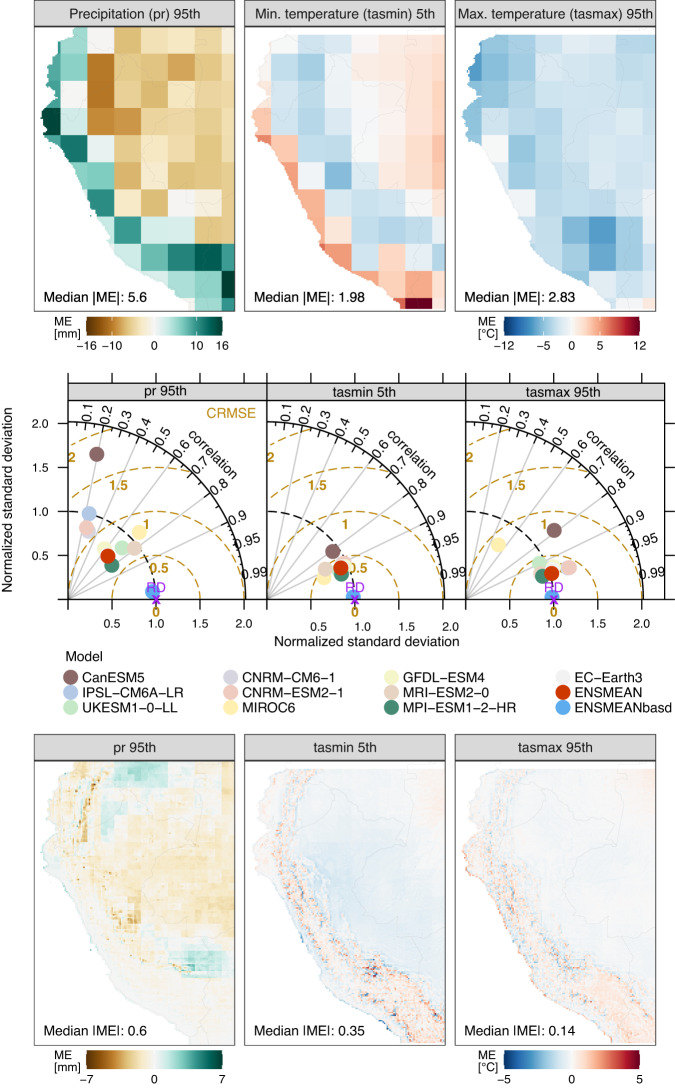
Mean Errors [ME] in (Top) unadjusted [ENSMEAN] and (Bottom) adjusted [ENSMEANbasd] CMIP6 multimodel ensemble means, and (Middle) Taylor diagrams, both comparing simulated and reference climate extremes for 1981–2014. Adjusted models were excluded from the Taylor diagrams as they closely match ENSMEANbasd and the reference data. The bottom panel displays ME in ENSMEANbasd, computed by comparing BASD-CMIP6-PE and observational data at a 0.1° spatial resolution.

The assessment of the models’ performance in simulating precipitation and temperature extremes, both before and after applying BASD, is shown in Fig. 5. In this figure, Taylor diagrams compare the degree of similarity in spatial patterns of extremes, considering their correlation, RMSE, and standard deviation.

Results show that CMIP6 models, particularly CanESM5, exhibit poor performance in simulating extreme values, as indicated by Taylor diagrams (Fig. 5, middle panel). Figure 5 (top panel) shows notable biases in the unadjusted multimodel ensemble (ENSMEAN), with distinct spatial patterns. ENSMEAN tends to overestimate extreme precipitation along the Andes while underestimating it over the Amazon lowland regions. Moreover, it exhibits a warm bias in extreme minimum temperatures in coastal areas and the Brazilian lowlands, while concurrently revealing a cold bias in the transitional zone between the Andes and the Amazon. A cold bias is also observed in extreme maximum temperatures across the entire study area.

These biases are substantially reduced by the adjusted multimodel ensemble (ENSMEANbasd) for all extreme climatic variables assessed herein, as demonstrated by Taylor diagrams (Fig. 5, middle panel) and the adjusted CMIP6 multimodel mean errors (Fig. 5, bottom panel). The results clearly show that the BASD method improves the variability and extremes of precipitation and temperature, thereby establishing the reliability of the BASD-CMIP6-PE dataset for studying the impacts of climate change on extreme events.

### Hydrological evaluation of unadjusted and adjusted CMIP6 models for the historical period

**Fig. 6:**
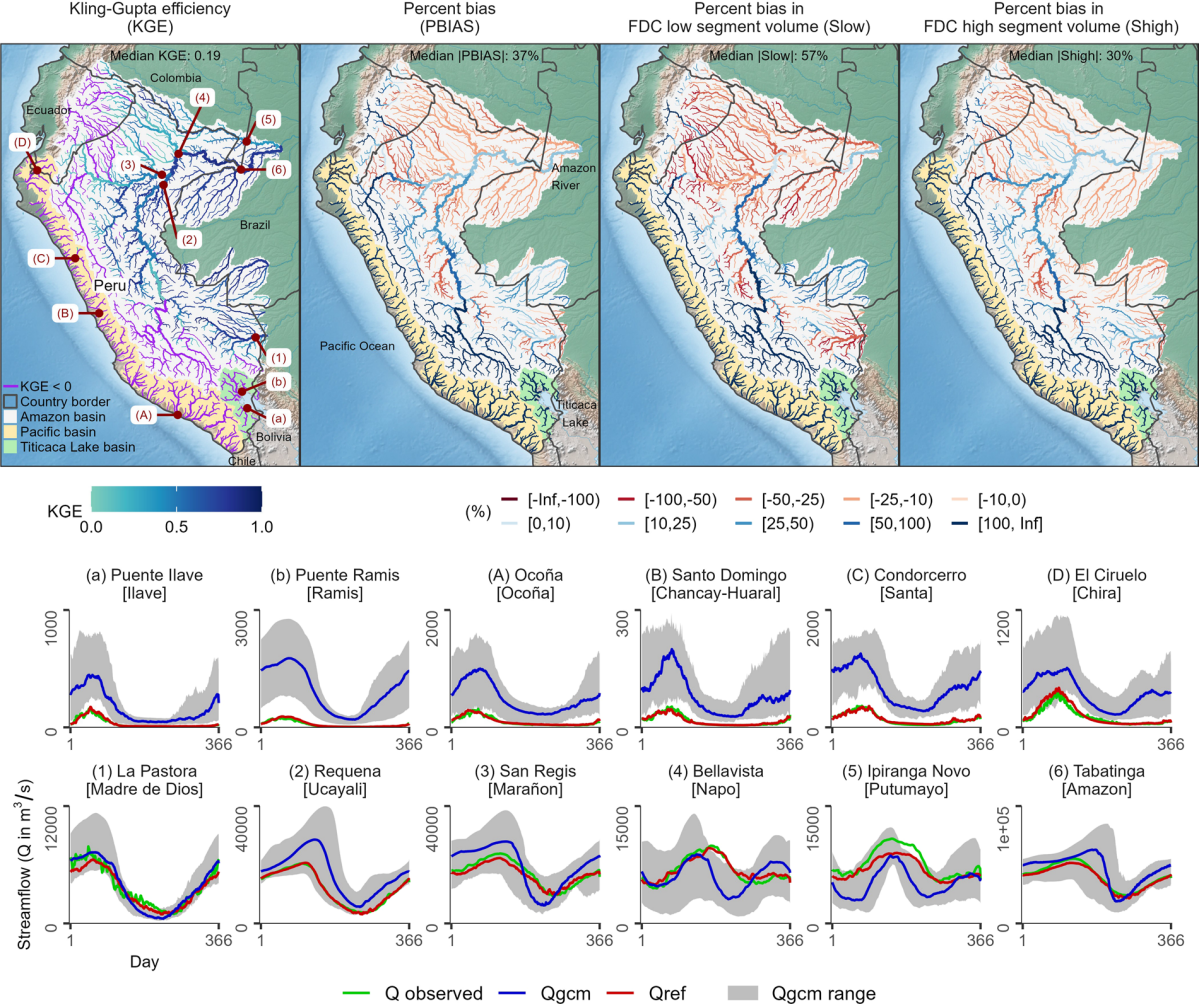
Comparison of simulated streamflow dynamics, including extreme events of both low flows (S_low_) and high flows (S_high_), from raw GCM simulations (Qgcm) and reference climate-based simulated streamflow (Qref). (top) Statistical metrics and hydrological signatures and (bottom) daily values of climatological seasonal streamflow (Q) in the period 1984–2014 for representative river catchments draining into the Titicaca Lake (a,b), the Pacific Ocean (A:D), and the Amazon River (1:6). Note that observed seasonal streamflow was computed only using the days with available streamflow data.

**Fig. 7:**
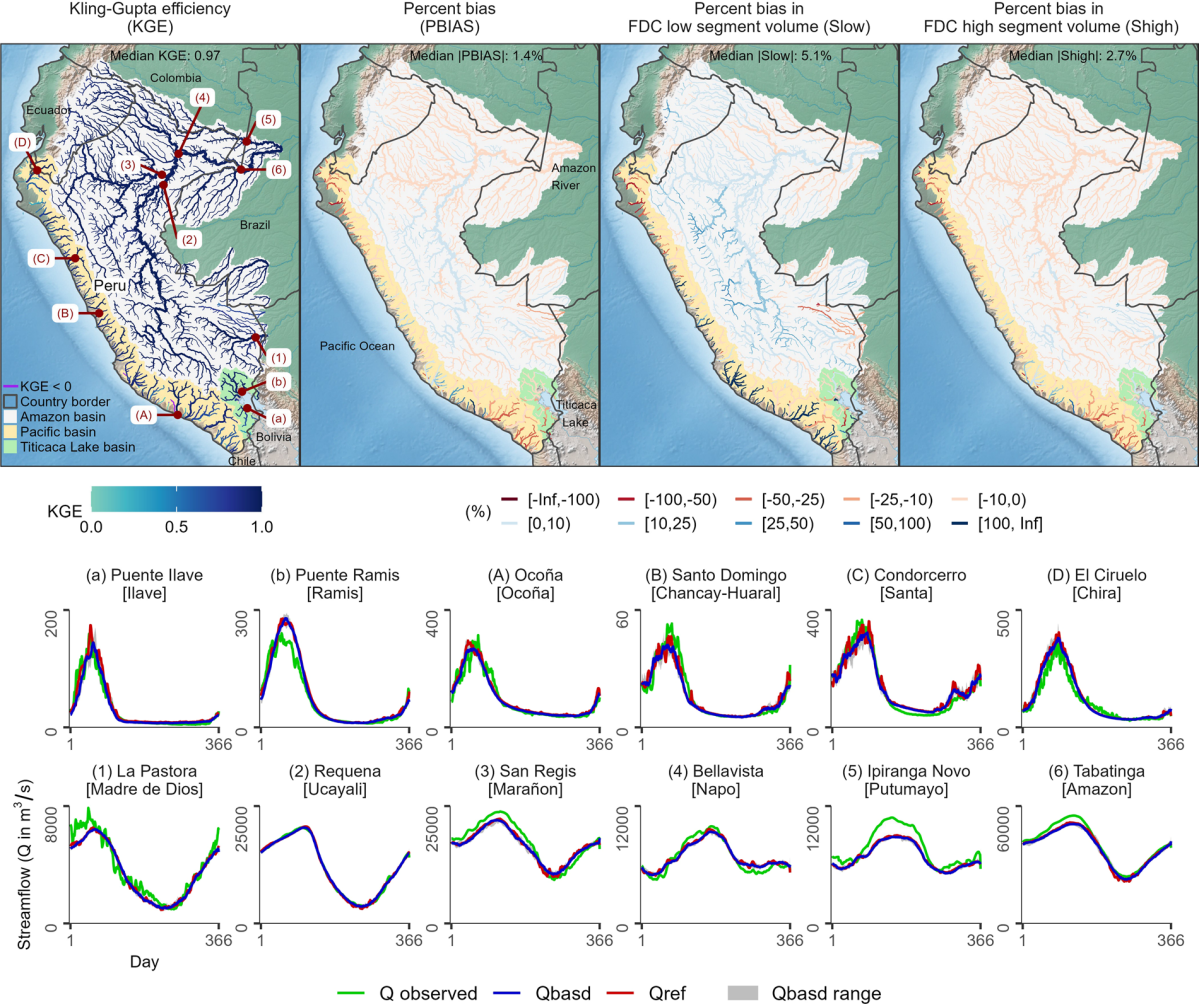
Comparison of simulated streamflow dynamics, including extreme events of both low flows (S_low_) and high flows (S_high_), from adjusted GCM simulations (Qbasd) and reference climate-based simulated streamflow (Qref). (top) Statistical metrics and hydrological signatures and (bottom) daily values of climatological seasonal streamflow (Q) in the period 1984–2014 for representative river catchments draining into the Titicaca Lake (a,b), the Pacific Ocean (A:D), and the Amazon River (1:6). Note that observed seasonal streamflow was computed only using the days with available streamflow data.

In Fig. 6 (or Fig. 7), a comparison is presented between the long-term mean annual streamflow cycle at a daily resolution derived from Qgcm (or Qbasd) and that derived from Qref. The comparison is made through statistical metrics (KGE and PBIAS) and hydrological signatures (S_low_ and S_high_). These figures also show climatological seasonal streamflow plots for representative river catchments draining into the Titicaca Lake, the Pacific Ocean, and the Amazon River.

In Fig. 6, the comparison metrics (with median values of KGE = 0.19, |PBIAS| = 37%, |S_low_| = 57%, and |S_high_| = 30%) alongside seasonal streamflow plots reveal significant discrepancies in the mean annual streamflow cycle between Qgcm and Qref. These disparities indicate that hydrological model simulations using raw GCM data tend to overestimate mean, low, and high flows along the Andean rivers while underestimating them over Amazonian lowland tributaries, especially in the northern Peruvian Amazon and Ecuadorian Amazon. Additionally, these simulations underrepresent seasonal streamflow in these regions, as evidenced by the seasonal plots for the Marañón, Napo, and Putumayo rivers. These issues underscore how biases and regional seasonal underrepresentation in GCM simulations impact the accurate representation of hydrological processes, rendering them unsuitable for hydrological impact assessments.

In Fig. 7, median KGE = 0.97 and median |PBIAS| = 1.4% demonstrate good agreement between Qbasd and Qref. This agreement is further supported by long-term mean seasonal streamflow plots for representative rivers across the three drainage systems. Low KGE (<0.5) and large negative PBIAS values (<−25%) indicate relatively poor BASD performance over northern and southern Peruvian arid coastal areas in the Pacific drainage system. It is worth noting that these areas have mean annual precipitation of less than 15 mm and are not relevant for runoff processes. Median |S_low_| = 5.1% and median |S_high_| = 2.7% also indicate that overall low and high flows are well represented, suggesting that the BASD method is able to represent also precipitation extremes to some extent. However, there is a tendency to overestimate low flows over river segments in the eastern slopes of the Peruvian Andes, especially over mountainous catchments.

Overall, the hydrological comparisons reveal much better agreement between Qbasd and Qref (median KGE = 0.97) compared to Qgcm and Qref (median KGE = 0.19) across different hydroclimate regimes in Peru and Ecuador. This indicates that the BASD method is effective, and the BASD-CMIP6-PE dataset is reliable for hydroclimatic applications, including extremes such as floods and droughts. However, it is important to keep in mind that the hydrological evaluation was performed for the period where the BASD method was trained, and our evaluation results could be overconfident. Nevertheless, we believe that our hydrological evaluation is still plausible in the study area featured by data scarcity and lack of long-term time series.

### Projected changes and inter-model spread

**Fig. 8:**
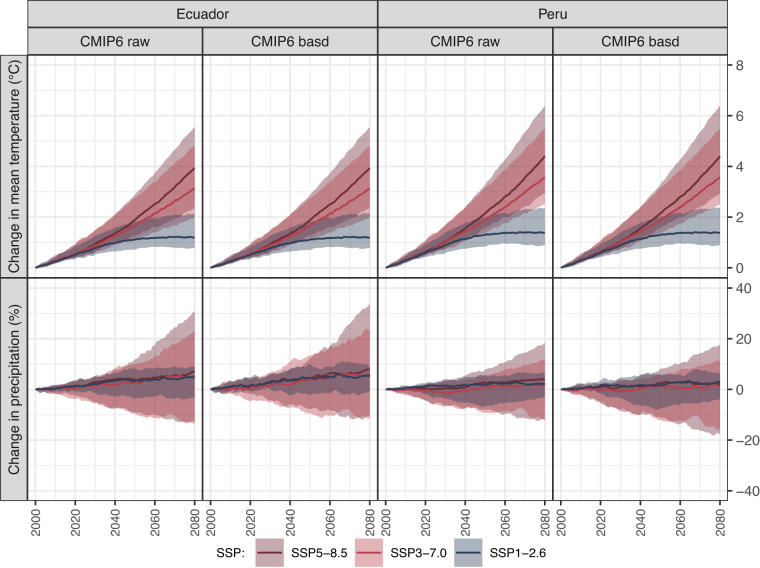
Comparison of projected multimodel median changes and spreads in precipitation and mean temperature for Ecuador and Peru using raw (CMIP6 raw) and adjusted (CMIP6 basd) GCM simulations.

The assessment of the BASD method’s impact on preserving or altering the projected GCM trends and the inter-model spread is presented in Fig. 8. This figure presents the projected multimodel median changes and spreads in precipitation and mean temperature for both Ecuador and Peru, using both unadjusted and adjusted CMIP6 data.

Results show that temperature trends and spreads show a significant degree of similarity between the two datasets in each country, highlighting the effectiveness of the BASD method in preserving future temperature trends. For Ecuador, the projected changes in mean temperature for the end of the century (2065–2095) relative to the reference period of 1985–2015, based on both raw and adjusted multimodel median data, are 1.2 °C (SSP1-2.6), 3.1 °C (SSP3-7.0), and 3.9 °C (SSP5-8.5). For Peru, these values are 1.4 °C (SSP1-2.6), 3.6 °C (SSP3-7.0), and 4.4 °C (SSP5-8.5).

However, the projected multimodel median changes and spreads for precipitation undergo minor modifications. The differences in projected changes for the end of the century, based on raw and adjusted CMIP6 data, are less than 2% across all SSPs for both Ecuador and Peru. These small differences suggest that the BASD method alters precipitation patterns without adversely affecting the ensemble median. Projected multimodel median changes in precipitation can range up to 8% in Ecuador and 3% in Peru, with variations within these countries.

It is noteworthy that intermodel spread increases towards the distant future, with low uncertainty under SSP1-2.6 and high uncertainty under SSP5-8.5.

In summary, our analysis indicates that the BASD method effectively preserves projected temperature trends while making only minor adjustments to precipitation patterns. This underscores the reliability of the BASD-CMIP6-PE dataset for evaluating regional and local hydrological impacts of climate change.

## Data Availability

The software used for bias adjustment and statistical downscaling is ISIMIP3BASD v2.5^29^.
